# Influence of Vitamin D on the Growth and Development of Uterine Fibroids

**DOI:** 10.7759/cureus.114365

**Published:** 2026-08-11

**Authors:** Noura E Awad

**Affiliations:** 1 Obstetrics and Gynecology, Ministry of Interior, Doha, QAT; 2 Obstetrics and Gynecology, Egyptian Medical Syndicate, Cairo, EGY; 3 Assisted Reproduction Technology, University of Kiel, Kiel, DEU

**Keywords:** classification, fibroids, molecular mechanisms, treatment, vitamin d

## Abstract

Uterine fibroids represent the most frequently encountered benign gynecological tumors in women of reproductive age. Key risk factors include vitamin D deficiency, obesity, dark skin pigmentation, low parity, premenopausal status, hereditary/hormonal backgrounds, and heavy alcohol consumption. Although benign, fibroids severely diminish the quality of life via symptoms such as abnormal uterine bleeding, pelvic pressure, urinary frequency/retention, anemia, infertility, and poor obstetric outcomes. Current surgical, medical, and interventional therapies impose immense financial burdens on health systems while carrying substantial procedural risks and drug side effects, necessitating safe alternative treatments.

A systematic literature search was conducted following PRISMA guidelines. PubMed Central (PMC) and Google Scholar were searched from inception through (July 2023 to 2025) using the following search string: ("Vitamin D" OR "cholecalciferol" OR "ergocalciferol" OR "1,25-dihydroxyvitamin D" OR "vitamin D deficiency") AND ("uterine fibroids" OR "uterine leiomyoma" OR "leiomyomata") AND ("in vitro" OR "in vivo" OR "animal model" OR "clinical trial" OR "epidemiology" OR "demographics").

Inclusion criteria comprised peer-reviewed human clinical trials, in vivo animal studies, and in vitro investigations evaluating vitamin D's relationship, molecular mechanisms, or therapeutic effects on uterine fibroids, including demographic disparities, published in English. Editorials, conference abstracts, narrative reviews, and studies focusing on other gynecological conditions were excluded.

Study selection involved duplicate removal, independent title/abstract screening, and full-text assessment, with disagreements resolved by consensus. Risk of bias was independently assessed using the Cochrane Risk of Bias (RoB) 2 and Newcastle-Ottawa Scale for human studies, Systematic Review Centre for Laboratory Animal Experimentation's (SYRCLE) tool for animal models, and tailored quality parameters for in vitro studies. Due to methodological heterogeneity, a qualitative narrative synthesis was performed rather than a meta-analysis.

Synthesized data confirm that uterine fibroid tissue is highly correlated with deficient vitamin D levels. Mechanistically, vitamin D exerts antiproliferative effects, mitigates cellular DNA damage, and modulates estrogen and progesterone receptor (PR) expression within fibroid cells. In vitro and in vivo animal models demonstrate the high efficacy of vitamin D in suppressing leiomyoma growth. Similarly, existing clinical trials support its role in regressing physical fibroid size and volume without causing significant adverse effects.

Vitamin D presents a safe, well-tolerated, and highly cost-effective therapeutic option for uterine fibroids, particularly for patients seeking fertility preservation or those deemed surgically unfit. While current data are highly promising and capable of reducing global healthcare burdens, large-scale randomized clinical trials (RCTs) and robust meta-analyses are urgently required. Expanding this research will establish definitive clinical guidelines regarding standardized dosing, treatment duration, and precise patient selection criteria based on age, symptoms, and surgical fitness.

## Introduction and background

Overview and etiology

Uterine fibroids, alternatively designated as myomas or leiomyomas, represent the most prevalent benign neoplasms of the female reproductive tract during the childbearing years. These tumors characteristically originate from the neoplastic proliferation of uterine smooth muscle cells (myometrium) [1].

Epidemiology and prevalence

The epidemiological burden of uterine fibroids varies notably across different demographics, with an estimated prevalence ranging from 70% in Caucasian populations to as high as 80% among women of African descent [2].

Clinical presentation

While a significant proportion of patients remain completely asymptomatic, approximately 30% present with clinically significant morbidity. Symptomatic individuals frequently experience severe manifestations, including menorrhagia (abnormal uterine bleeding), secondary iron-deficiency anemia, and pelvic bulk symptoms. These pressure-related effects encompass pelvic pain or heaviness, chronic low back pain, constipation, and urinary frequency. Furthermore, fibroids are clinically associated with reproductive challenges, including unexplained infertility, recurrent miscarriages, and adverse obstetric outcomes [3].

Classification systems

Anatomically, fibroids have historically been categorized based on their structural relationship to the endometrium and the uterine serosa into three primary groups: intramural, subserosal, and submucosal, with intramural variants being the most frequently diagnosed [4].

**Figure 1:**
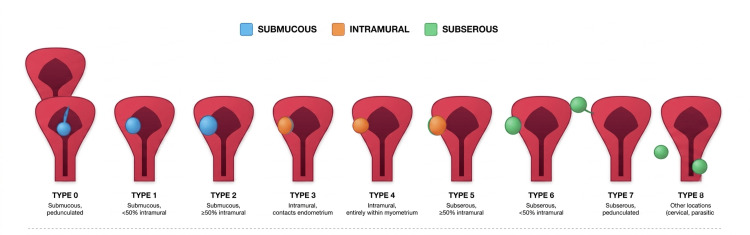
Different types of fibroids according to FIGO classification. FIGO classification of uterine fibroids: 0 = pedunculated intracavitary fibroid, 1 = submucosal fibroid < 50% intramural, 2 = submucosal fibroid ≥ 50% intramural, 3 = 100% intramural but in contact with the endometrium, 4 = intramural fibroid, 5 = subserosal fibroid that is ≥50% intramural, 6 = subserosal fibroid that is <50% intramural, 7 = subserosal pedunculated fibroid, 8 = other (e.g., cervical and parasitic) fibroids, and 2-5 = hybrid fibroid that is <50% submucosal and <50% subserosa. This figure was created by the author as an illustration to explain the FIGO classification system for uterine fibroids. The image was created using Microsoft Word (Microsoft Corp., Redmond, WA) and Adobe Illustrator (Adobe Inc., Mountain View, CA). FIGO: International Federation of Gynecology and Obstetrics

To standardize diagnosis and management, the International Federation of Gynecology and Obstetrics (FIGO) introduced a comprehensive nine-tier classification system (Types 0-8) in 2011 [5]. This mapping operates as follows: Type 0, entirely pedunculated and intracavitary; Type 1, submucosal, with an intramural extension of less than 50%; Type 2, submucosal, with an intramural extension equal to or greater than 50%; Type 3, entirely intramural but directly contacting the endometrium; Type 4, purely intramural, confined entirely within the myometrium; Type 5, subserosal, with an intramural component equal to or greater than 50%; Type 6, subserosal, with an intramural component of less than 50%; Type 7, pedunculated and subserosal; Type 8, ectopic or atypical locations (e.g., cervical or parasitic fibroids); and Types 2-5 (hybrid), transmural lesions that are less than 50% submucosal and less than 50% subserosal (Figure 1) [5].

In 2018, the FIGO classification was updated to include uterine volume, the number of fibroids, and the volume of up to four fibroids in the ultrasound report, in addition to the relationship of the fibroids to the endometrium and their location [6].

Risk factors

Hormonal Influences

Extensive clinical data link hormonal patterns, such as early menarche (first menstruation), late menopause, low parity (fewer childbirths), and rapid tumor enlargement during pregnancy, to fibroid development. These patterns underscore the critical roles of estrogen and progesterone. Because both hormone levels drop following menopause, fibroids typically regress during this stage.

While uterine fibroids were historically viewed as strictly estrogen-dependent tumors [7,8], recent evidence highlights that progesterone and its receptors are also primary drivers of fibroid growth [9]. Furthermore, estrogen facilitates this process by mediating the expression of progesterone receptors (PR), enabling progesterone to act on target cells [10]. However, the precise functional interplay between estrogen and progesterone in fibroids is still being actively investigated [11].

Age

The risk of developing uterine fibroids increases notably in premenopausal women between the ages of 35 and 39 [12,13]. Conversely, the condition is exceptionally rare in adolescents [13].

Obesity

Women with obesity face a higher susceptibility to uterine fibroids [14,15], with the risk escalating in direct proportion to every kilogram of excess body weight [16,17]. A comprehensive analysis of 22 studies by Parazzini et al. firmly established this association between obesity and increased fibroid risk [18].

Racial Disparities

Uterine fibroids occur more frequently in Black women [19]. This increased prevalence may be tied to higher systemic exposure to stress [20], discrimination [21], and vitamin D deficiency [22]. Other contributing variables include higher rates of obesity, lower fruit intake [13], elevated concentrations of steroid hormones, and distinct genetic polymorphisms, such as those affecting the catechol-O-methyltransferase (*COMT*) gene [23]. Additionally, fibroid risk in African American women has been specifically linked to two single-nucleotide polymorphisms (SNPs): rs1280043 near the 7-dehydrocholesterol reductase (*DHCR7*) gene and rs6058017 within the agouti-signaling protein (*ASIP*) gene [24].

Parity

There is an inverse relationship between childbirth and fibroid development [25]. The statistical risk of developing uterine fibroids declines progressively with each subsequent pregnancy [26].

Hypertension

Women with high blood pressure experience a fivefold increase in fibroid risk compared to those with normal blood pressure [27]. An earlier diagnosis of hypertension correlates with an even higher risk of fibroid development; this may be due to increased blood flow causing myometrial (uterine muscle) injury, which subsequently triggers the release of inflammatory cytokines [28].

Lifestyle and Substance Use

Heavy alcohol consumption correlates with an elevated risk of developing fibroids [29]. Conversely, the impact of tobacco smoking remains a subject of debate [30]. Some research indicates an inverse relationship where smoking appears to lower the risk of fibroids, potentially because cigarette use can reduce circulating estrogen levels [31]. However, conflicting data suggest that certain components of cigarette smoke might actually amplify estrogenic effects on the uterus, thereby stimulating cell proliferation [32].

Vitamin D Deficiency

Contemporary research identifies vitamin D deficiency as a distinct risk factor, consistently linking low vitamin D levels to accelerated uterine fibroid growth [33-35].

## Review

Vitamin D metabolism

Vitamin D, also known as calciferol, is a fat-soluble vitamin. It is naturally present in a few foods, such as fatty fish, milk, egg yolk, meat, liver, and some fortified cereals. Vitamin D is also synthesized endogenously in the presence of sunlight, particularly ultraviolet (UV) rays. Most people meet their daily requirements through sunlight exposure [36]. Vitamin D has two main forms: D2 (ergocalciferol) and D3 (cholecalciferol). It is absorbed mainly in the small intestine by passive diffusion and intestinal membrane carrier proteins [37]. Both exogenous and endogenous forms of vitamin D are biologically inert and undergo two hydroxylation steps. The first occurs in the liver, forming 25-hydroxyvitamin D (25(OH)D), also known as calcidiol. The second occurs in the kidney, resulting in 1,25-dihydroxyvitamin D (1,25(OH)2D), also known as calcitriol [38].

A serum 25-hydroxyvitamin D (25(OH)D) level of >20 ng/mL is considered adequate for general health [39]. Although 25(OH)D is a biomarker of exposure, the extent to which 25(OH)D levels relate to health status or outcomes remains unclear [40]. There is insufficient evidence to support routine screening in asymptomatic adults [41].

Risk factors for vitamin D deficiency

Many factors may place women at risk of vitamin D deficiency. Obesity can interfere with the skin synthesis of vitamin D, and bariatric surgery can interfere with vitamin D absorption [42]. Women with ulcerative colitis, Crohn's disease, liver disease, and cystic fibrosis may also have impaired vitamin D absorption and synthesis [43]. Dark skin can reduce cutaneous vitamin D synthesis [44]. Women who remain indoors, have limited sun exposure, or wear covering clothing for religious reasons may also be at increased risk [40]. In addition, vitamin D synthesis in the skin decreases with aging [45]. Vitamin D deficiency mainly affects bone health, causing rickets in children and osteoporosis in adults [46]. Therefore, breastfeeding mothers are advised to use vitamin D supplements, as breast milk is poor in vitamin D [47]. Some experts have noted that vitamin D deficiency may play a role in chronic diseases, such as diabetes, cardiovascular disease, neuropsychiatric disorders, autoimmune diseases, and tumors [48]. Experts now also consider vitamin D deficiency a possible contributor to uterine fibroid growth [33-35]. In 2013, Baird et al. found that the risk of uterine fibroids in women with normal serum vitamin D levels was 32% lower than in women with vitamin D deficiency [33].

Paffoni et al. studied 128 Italian women with uterine fibroids and divided them into three groups: vitamin D deficient (<10 ng/mL), insufficient (10-19.9 ng/mL), and sufficient (≥20 ng/mL). They compared these women to a control group of 256 healthy women and found that women with uterine fibroids had significantly lower vitamin D levels than the healthy controls [35]. Significantly lower vitamin D levels in women with uterine fibroids compared with women with normal vitamin D levels were also reported by Li et al. in a study conducted in 2020 [49]. A study from East India confirmed that women with uterine fibroids had lower serum vitamin D levels than the control group but found no correlation between vitamin D level and disease extent [50]. Mohammadi et al. summarized data from a meta-analysis and concluded that low vitamin D levels are associated with a higher incidence of uterine fibroids [51]. Some studies have found a correlation between vitamin D deficiency and uterine fibroids in Black women, as vitamin D deficiency is reported to be 10 times higher in Black women [22].

In 2022, Guo et al. conducted the first Mendelian randomization study based on summary data from the UK Biobank and concluded that reduced serum vitamin D3 was associated with an increased risk of uterine fibroids [52].

Cellular origin and molecular biology behind the development of uterine fibroid

A genetic background may contribute to uterine fibroids, as in most tumors, through somatic mutations and germline alterations. Driver mutations in the transcriptional mediator complex subunit 12 (*MED12*) gene in uterine myometrial cells initiate approximately 70% of uterine fibroids that grow in a progesterone-dependent manner. The treatment of primary mutant-*MED12* fibroid cells with tryptophan or kynurenine stimulated aryl hydrocarbon receptor (AHR) nuclear translocation, increased proliferation, inhibited apoptosis, and induced AHR-target gene expression, while blocking the tryptophan 2,3-dioxygenase (TDO2)/kynurenine/AHR pathway by siRNA abolished these effects. Progesterone receptors regulated the expression of AHR and its target genes. In vivo, TDO2 expression positively correlated with the expression of genes crucial for fibroid growth [53,54]. In addition, *MED12* mutations have been linked to pathways involved in uterine fibroid development, including the wingless-type (Wnt)/beta-catenin, protein kinase B/mammalian target of rapamycin (AKT/mTOR), focal adhesion, extracellular matrix (ECM), angiogenic, and hypoxia-inducible factor 1-alpha (HIF1α) pathways [55-58]. *MED12* mutations have also been noted in small fibroids and in tumors with an increased number [55], and they have been linked to the subserosal type of fibroid while being inversely related to parity [59].

Chromothripsis in uterine fibroids leads to chromosomal rearrangements, sometimes with numerous deletions and multiple breakpoints affecting limited genomic regions in only one of the homologous chromosomes [53]. The cause of this type of rearrangement is still poorly understood [60]. Nearly 50% of fibroids have recognizable cytogenetic rearrangements, such as deletions of 7q and rearrangements involving 12q15 or 6p21 [61]. These occur in 17% and 20% of cases, respectively, and in 5% of karyotypically abnormal lesions. *HMGA2*, which encodes high-mobility group AT-hook 2, is the driver gene for tumors carrying 12q15 rearrangements [62,63]. Chromosomal band 14q24 is the *HMGA2* translocation partner in fibroids [62]. Rearrangements affecting *HMGA1*, which encodes high-mobility group AT-hook 1, at 6p21 are also noted and include 14q24 in some cases [64,65]. The 14q24 breakpoint maps to the RAD51B locus [66]. RAD51 homologue B has a role in the repair of DNA double-strand breaks through homologous recombination [67]. The second most common chromosomal change in fibroids is an interstitial deletion within 7q [62].

Carbajo-García et al. explained that the process of uterine fibroid development is controlled by DNA methylation. The inhibition of DNA methyltransferases by 5-aza-2'-deoxycytidine increased the expression of hypermethylated and downregulated genes in uterine fibroid cells in vitro. Based on this, they suggested that reversing DNA methylation involved in uterine fibroid development could be a promising future treatment [68].

Some studies show evidence of inheritance, especially among first-degree relatives [69].

**Figure 2:**
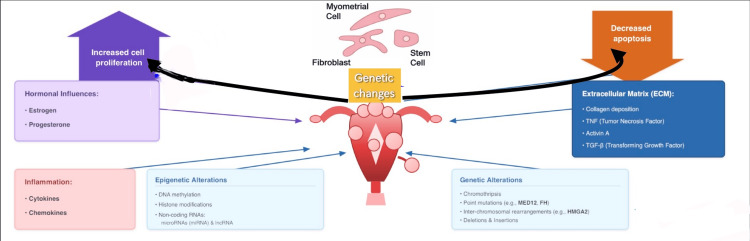
Different mechanisms that are involved in the growth of uterine fibroids. This figure describes different mechanisms that are involved in the growth of uterine fibroids (UFs), including cellular mechanisms in the extracellular matrix (ECM), decreased apoptosis in stem cells with increased cell proliferation in myometrial cells, hormonal influence on UF development, gene mutation, and epigenetics alteration's role in UF development. This figure was created by the author to simplify the different mechanisms that are involved in the development of uterine fibroids. The image was created using Microsoft Word (Microsoft Corp., Redmond, WA) and Adobe Illustrator (Adobe Inc., Mountain View, CA).

The cellular origin of uterine fibroids involves monoclonal cells. Under specific environmental and genetic changes, myometrial stem cells may undergo reduced apoptosis and uncontrolled proliferation, leading to fibroid development [70]. Cells with stemlike characteristics have also been isolated from uterine fibroids and myometrium [71,72]. Uterine fibroids are characterized by excessive collagen deposition in a disorganized extracellular matrix (ECM), as well as growth factors, including transforming growth factor-beta (TGF-beta), activin-A, and platelet-derived growth factor; cytokines, including tumor necrosis factor-alpha (TNF-alpha); and steroid hormones, including estrogen and progesterone [73,74]. MicroRNA also plays a role in ECM accumulation [75]. Chronic inflammation caused by factors such as uterine infection, menstruation, injury, and obesity may contribute to uterine fibroid development and growth through the release of inflammatory mediators, including cytokines and chemokines, which have been detected in uterine fibroid specimens (Figure 2) [76].

Management of uterine fibroid

Diagnosis of Uterine Fibroid

Uterine fibroids can be diagnosed through proper history taking, the evaluation of the patient's symptoms, assessment for signs of anemia, and clinical examination. During bimanual pelvic examination, they may be felt as an irregular mass in a mobile uterus [77]. Sonography is the first-line imaging modality for uterine fibroids, and saline infusion may be added when a submucosal fibroid is suspected [78]. If saline sonography is inconclusive, MRI is highly recommended, especially before proceeding to myomectomy or hysteroscopy. MRI can also be used for submucosal fibroids when biopsy or resection is planned or when malignancy is suspected [79].

Treatment options for uterine fibroids

Expectant Management

Expectant management is reserved for asymptomatic premenopausal women, as uterine fibroids may regress within six months to three years [80].

Surgical Treatment

Surgery remains the main treatment for symptomatic uterine fibroids and includes hysterectomy and myomectomy. Uterine fibroids are the leading cause of hysterectomy in the United States [81].

Hysterectomy is considered the definitive treatment for symptomatic women who do not wish to preserve fertility [82]. It can be performed vaginally, by laparoscopic-assisted vaginal hysterectomy, or abdominally. Vaginal hysterectomy is preferred because it is associated with a shorter hospital stay and less blood loss, although its use is limited by fibroid size and patient selection [83]. Abdominal hysterectomy is an alternative for patients who are not suitable candidates for vaginal hysterectomy; however, it carries a higher risk of bleeding, infection, urinary tract or bowel injury, paralytic ileus, thrombosis, pulmonary embolism, and, rarely, death. Nearly one in 10 women may develop new symptoms after hysterectomy with bilateral salpingo-oophorectomy [84].

The laparoscopic morcellation of uterine fibroids is a concern because of the possibility of occult malignancy [85]. The American College of Obstetricians and Gynecologists advised that this technique should not be used in women with suspected or known malignancy and emphasized the importance of informed preoperative consent [86]. Myomectomy may be offered to women who wish to preserve fertility, and it can be performed laparoscopically, abdominally, or hysteroscopically. Laparoscopic myomectomy is preferred because it is associated with less postoperative pain, a shorter hospital stay, and less blood loss than open myomectomy [87]. Hysteroscopic myomectomy is reserved for submucosal fibroids smaller than 3 cm when more than 50% of the fibroid protrudes into the uterine cavity [88]. The recurrence rate after myomectomy is approximately 15%-33%, and almost 10% of women who undergo the procedure will require hysterectomy within 10 years [77].

Interventional Treatment

Uterine artery embolization is a conservative interventional treatment and has become a major second-line option for selected patients who wish to preserve the uterus or who cannot tolerate surgery [89]. The most common complication is post-embolization syndrome, which includes pain, fever, and fibroid expulsion. In addition, uterine artery embolization is contraindicated in renal insufficiency, contrast allergy, and pelvic infection. Future fertility after uterine artery embolization remains uncertain, and further studies are needed [90].

More recent treatments use minimally invasive energy-focused devices, such as laser, heat-based techniques, and magnetic resonance-guided focused ultrasound surgery (MRgFUS), to lyse fibroids. Although these approaches are minimally invasive and generally well-tolerated, they carry risks of localized pain and bleeding [91], and their effects on future fertility require further investigation [92].

Medical Treatment

Several medical options may be offered to patients according to their treatment goals, including combined oral contraceptive pills (COCPs), luteinizing hormone-releasing hormone (LHRH) agonists, progestins, the levonorgestrel intrauterine system (LNG-IUS), selective estrogen receptor (ER) modulators, gonadotropin-releasing hormone (GnRH) agonists, and selective progesterone receptor modulators (SPRMs). GnRH agonists and SPRMs have the strongest evidence for symptom control because they shrink fibroid size and decrease menstrual blood loss [93]. Combined hormonal contraception significantly decreases menstrual blood loss in patients with uterine fibroids [94]. The progesterone intrauterine system (Mirena) provides greater control of menstrual blood loss; however, the expulsion rate may reach up to 20% in patients with uterine fibroids [95].

Tranexamic acid is an antifibrinolytic agent that significantly controls menstrual blood loss [96]; however, some studies have reported fibroid necrosis in the treatment group compared with patients who received no treatment [97].

Nonsteroidal anti-inflammatory drugs can also be used to control menstrual blood loss and reduce pain, but they are less effective than Mirena [98].

Gonadotropin-releasing hormone (GnRH) agonists and selective progesterone receptor modulators (SPRMs) are good options for women approaching menopause who need temporary symptom relief. GnRH agonists are used preoperatively to decrease blood loss and shrink tumor size. Treatment is limited to three months to avoid adverse effects on bone density caused by hypoestrogenism [99]. Although SPRMs have the advantage of lacking hypoestrogenic effects on bone loss, benign endometrial changes have been noted [100].

Aromatase inhibitors and estrogen receptor antagonists require further study, as their risks may outweigh their benefits [101].

Vitamin D as a treatment option for uterine fibroid

The main role of vitamin D is to regulate calcium and phosphorus metabolism in order to maintain healthy bones and adequate muscle function. Vitamin D also has other roles in the body, including supporting immune function, glucose metabolism, the regulation of cell growth, proliferation and apoptosis, and anti-inflammatory activity [102]. In addition, vitamin D may alter the molecular biology of uterine fibroids [103]. The link between vitamin D and uterine fibroid pathology has led researchers to investigate vitamin D as a possible treatment option for uterine fibroids [33-35].

Vitamin D and fibroid in vitro studies

Uterine fibroids contain low levels of 1,25-dihydroxyvitamin D3 and show dysregulated expression of vitamin D-metabolizing enzymes [104]. In one study performed by Bläuer et al., vitamin D showed a dose-dependent antiproliferative effect on fibroid cells [105]. Sharan et al. showed that vitamin D treatment reduced uterine fibroid growth in association with the reduced expression of proliferating cell nuclear antigen (PCNA), B-cell lymphoma 2 (Bcl-2), Bcl-w, cyclin-dependent kinase 1 (CDK1), and catechol-O-methyltransferase (COMT) [106]. Reis et al. suggested that vitamin D has a regulatory role in estrogen signaling, which may contribute to uterine fibroid growth through the overexpression of estrogen and progesterone receptors in fibroids compared with normal myometrium [107]. In myometrial fibroid cells, vitamin D reduces nuclear ERα, PR-A, and the expression levels of steroid receptor coactivator (SRC) 1, SRC2, and SRC3, emphasizing the antiestrogenic and antiprogesteronic role of the vitamin D receptor (VDR) [108]. In the myometrium, vitamin D modulates the expression of inflammatory markers and ERα when cocultured with human monocyte lineage (THP1) cells [109].

The effects of vitamin D on the wingless-type (Wnt)/beta-catenin pathway were investigated by Ono et al. [110]. Moreover, vitamin D treatment suppressed the Wnt/beta-catenin downstream signaling pathway mTOR. In an in vitro study by Corachán et al., VDR-knockdown cells with reduced VDR levels showed higher levels of phosphorylated mTOR and increased ECM protein expression [111]. Corachán et al. suggested that vitamin D could be introduced as a therapy for uterine fibroids by inhibiting Wnt/beta-catenin signaling. Using a PCR array in human uterine fibroid primary cells, they found that a large set of Wnt-related genes was modulated after vitamin D treatment. VDR activation inhibits the activation of Wnt-related genes involved in the regulation of biological processes such as tissue polarity, cell cycle, migration, proliferation, and growth [111].

Corachán et al. demonstrated that vitamin D expresses its antiproliferative action irrespective of *MED12* status. They studied the effects of vitamin D on the Wnt/beta-catenin and TGF-beta signaling pathways in human uterine fibroid cells isolated from *MED12*-mutated and wild-type tumors. In fact, Wnt/beta-catenin and TGF-beta pathway genes were increased in *MED12*-mutated fibroids [112].

An in vitro study showed that vitamin D causes the upregulation of VDR in cultured fibroid cells, although the molecular mechanisms behind this effect were not fully explained. In the same study, vitamin D treatment was associated with a significant reduction in the protein expression of ECM-associated collagen type I, fibronectin, and plasminogen activator inhibitor-1 (PAI-1). In addition, the mRNA and protein expression of proteoglycans such as fibromodulin, biglycan, and versican were reduced in human uterine fibroid cells. The study also showed that vitamin D decreased the deposition of disorganized smooth muscle actin fibers through the mRNA and protein downregulation of matrix metalloproteinase-2 (MMP-2) and MMP-9, while the expression of tissue inhibitors of matrix metalloproteinase-2 (TIMP-2) was increased by vitamin D [113].

Ali et al. reported that vitamin D decreases DNA damage and induces VDR expression. This may increase the rate of mRNA synthesis, as demonstrated by the significant induction of VDR transcripts [114]. Elkafas et al. observed that vitamin D reduces DNA damage load in myometrial stem cells exposed to the endocrine-disrupting chemical diethylstilbestrol [115].

Vitamin D and fibroid in vivo animal studies

Vitamin D deficiency was associated with a pre-fibroid state in the myometrium of female mice, with increased expression of sex steroid receptors, increased expression of proliferation-related genes, increased DNA damage, and fibrosis. It also enhanced inflammation and immunosuppression through regulatory T-cell expansion in murine myometrium compared to the control group [116].

In Eker rats, a preclinical study investigated the effect of oral vitamin D supplementation. This animal model and its derived cell lines share some phenotypic and biochemical characteristics with human uterine fibroids, including estrogen and progesterone receptors and responses to steroid hormones. Treated rats showed inhibition of tumor growth and cell proliferation through the downregulation of PCNA, cyclin D1, c-Myc, CDK1, CDK2, and CDK4 proteins. They also showed a reduction in tumor size. This may be related to apoptosis induction, confirmed by reductions in Bcl-2, Bcl-xL, ERα, PR-A, and PR-B after vitamin D supplementation [117]. Another in vivo experiment studied vitamin D and its analogue, and both showed a reduction in uterine fibroid volume in nude mice implanted with Eker rat tumor-derived Eker leiomyoma tumor-3 (ELT-3) cells compared with an untreated control group [118].

One study evaluated vitamin D treatment for uterine fibroids. Human fibroids from patients were implanted in ovariectomized nonobese diabetic-severe combined immunodeficient (NOD-SCID) mice and then treated with vitamin D (0.5 μg/kg/day or 1 μg/kg/day) or vehicle for 21 or 60 days. The study showed that long-term vitamin D therapy induced regression in fibroid size, as it acted as an antiproliferative, antifibrotic, and proapoptotic agent. In contrast, the short-term course of vitamin D did not show any regression in uterine fibroids [119].

Vitamin D and uterine fibroid clinical trials

Because vitamin D has shown promising results in both in vitro and in vivo animal models, more recent clinical studies have focused on vitamin D as a potential treatment for uterine fibroids that may reduce the need for surgery. An open-label clinical trial of vitamin D was carried out by Ciavattini et al. They selected 108 women with "small-burden" uterine fibroids and vitamin D deficiency and divided them into two groups: a treatment group that received vitamin D (50,000 IU per week for eight weeks and then 2,000 IU per day for 12 months) and an untreated control group. The treated group showed regression in uterine fibroid growth and a lower rate of surgical or medical treatment compared to the control group. In contrast, the control group showed uterine fibroid growth and an increased need for surgery due to symptom severity [120].

In a prospective, double-blind study of 69 women with uterine fibroids and vitamin D deficiency, participants were divided into two groups: treatment and placebo. The treatment group received an oral dose of 50,000 IU of vitamin D every two weeks for a total of 10 weeks. Six months after treatment, there was a significant increase in vitamin D levels, in addition to a significant reduction in uterine fibroid volume in the treated group compared to the control group [121].

In a randomized clinical trial (RCT), women with uterine fibroids and vitamin D deficiency received either 50,000 IU of oral vitamin D or a placebo for 12 weeks. There was no change in uterine fibroid volume after treatment; however, vitamin D appeared to block further fibroid tumor growth [122].

Ciebiera et al. followed two women aged 37 and 49 years who had symptoms of uterine fibroids. They were treated with a combination of vitamin D and ulipristal acetate (UPA). Both patients received daily treatment for three months with 7,000 IU vitamin D and 5 mg UPA. The first woman showed a significant reduction in symptoms, including pain, pressure, and urinary frequency, in addition to a 47.8% reduction in fibroid volume. In the second woman, most symptoms disappeared, and fibroid volume regressed by 63.3%. Because this study used a combination of two drugs, the efficacy of vitamin D alone was not properly tested [123].

Porcaro et al. designed a pilot study in which 30 women with symptomatic uterine fibroids were divided into two groups: treatment and control. Fifteen women were treated with vitamin D in combination with epigallocatechin-3-gallate (EGCG) and vitamin B6 for four months. The results showed that uterine fibroid volume decreased by 34.7% in the treated group and increased by 6.9% in the control group. The authors claimed that this combination could represent a new non-hormonal treatment option for women with uterine fibroids [124].

Recently, Orive et al. carried out an ambispective observational study of 20 women under 45 years of age with uterine myomas. The treatment group received low-dose vitamin D at 2,500 IU every two weeks, while the control group received no treatment. Alanine transaminase (ALT), aspartate transaminase (AST), bilirubin, calcium, and creatinine were measured at zero, three, and six months. At the beginning of the study, there was no significant difference between the two groups. At the end of the study, the treatment group showed a significant increase in vitamin D levels. Regarding fibroid size, the treatment group showed a lower growth rate compared to the control group (5.8% versus 17.3%). No significant changes in serum parameters, including calcium, ALT, AST, and creatinine, were observed in the treatment group [125].

More recent systematic review evidence suggests that the clinical effect of vitamin D supplementation is promising but not yet definitive. In 2024, a systematic review and meta-analysis included three randomized controlled trials and 23 observational studies and found that women receiving oral vitamin D supplementation had a statistically significant reduction in fibroid size compared to controls over 2-6 months, although heterogeneity was high. The review also found that women with fibroids had lower serum vitamin D concentrations and higher odds of vitamin D deficiency than women without fibroids. These findings strengthen the rationale for vitamin D as a possible noninvasive treatment strategy, but they also highlight the need for larger, standardized randomized trials with consistent dosing, duration, ultrasound measurement methods, and symptom-based outcomes [126].

**Figure 3:**
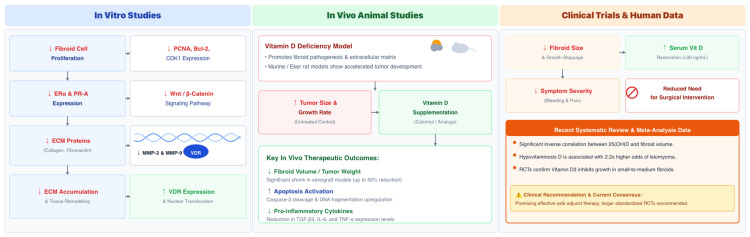
Summary of study results on vitamin D as a treatment option for uterine fibroids. Summary of different study results (in vivo, in vitro, and clinical trials), as in vivo and in vitro studies documented that uterine fibroid development is directly proportional to vitamin D deficiency; clinical trials that used vitamin D as treatment for UFs show a reduction in size and volume of fibroids. Meta-analysis shows more promising results. Blue color, in vitro studies; green color, in vivo studies; orange color, clinical trials and meta-analyses. This figure was created by the author as an illustration to highlight vitamin D as a treatment option for uterine fibroids. The image was created using Microsoft Word (Microsoft Corp., Redmond, WA) and Adobe Illustrator (Adobe Inc., Mountain View, CA). UFs, uterine fibroids; ECM, extracellular matrix; Bcl-2, B-cell lymphoma 2; PCNA, proliferating cell nuclear antigen; Wnt, wingless-type; MMP-2, matrix metalloproteinase-2; VDR, vitamin D receptor; TGF-β3, transforming growth factor-beta-3; TNF-α; tumor necrosis factor-alpha; RCTs, randomized clinical trials; 25(OH)D, 25-hydroxyvitamin D

Figure 3 shows vitamin D as a treatment option for uterine fibroids.

Future perspectives

Future research should include meta-analyses that combine data from small studies, as well as large-scale clinical trials, to establish clear clinical guidelines. These guidelines should address selection criteria for women with uterine fibroids who may be suitable for vitamin D treatment, appropriate patient age groups, fibroid-related symptoms that may respond to vitamin D, and the standard dose and duration of treatment. Further research is also needed to clarify the molecular biology behind uterine fibroid growth and how vitamin D deficiency affects the molecular and cellular biology of the myometrium.

## Conclusions

Uterine fibroids remain a widespread gynecological condition that presents a substantial burden on women's health and clinical management. Because they present with a wide range of symptoms, clinicians should consider several factors when planning treatment, including the patient's age, symptom severity, fertility preservation, and treatment-related morbidity. Although surgery remains the most effective treatment for symptomatic patients, many studies have attempted to highlight the molecular mechanisms of fibroid development. Recent studies have linked vitamin D deficiency to the molecular mechanisms of fibroid development. This relationship between vitamin D deficiency and uterine fibroid growth has led researchers to investigate vitamin D as a non-hormonal treatment option, with the additional benefit of increasing vitamin D levels in deficient women. No significant side effects have been recorded with this treatment, and it may also offer cost-effectiveness benefits compared to other treatment options.

Animal studies have shown promising results, with significant reductions in uterine fibroid size. Through the regulation of gene expression, vitamin D may mediate some of these effects by modulating intracellular signaling pathways, suggesting that vitamin D may be connected to multiple cellular processes. The clinical trials reviewed in this paper reported potentially effective treatment of uterine fibroids using vitamin D. However, the sample sizes were small, making the role of vitamin D in treating women with severe symptoms uncertain.
